# Stratification of Latent *Mycobacterium tuberculosis* Infection by Cellular Immune Profiling

**DOI:** 10.1093/infdis/jix107

**Published:** 2017-02-28

**Authors:** Alice Halliday, Hilary Whitworth, Sherine Hermagild Kottoor, Umar Niazi, Sarah Menzies, Heinke Kunst, Samuel Bremang, Amarjit Badhan, Peter Beverley, Onn Min Kon, Ajit Lalvani

**Affiliations:** 1 Tuberculosis Research Centre, Respiratory Medicine, National Heart and Lung Institute, Imperial College London, St Mary’s Campus,; 2 National Institute for Health Research, Health Protection Research Unit in Respiratory Infections, Imperial College London,; 3 Queen Mary University, and; 4 St Mary’s Hospital, Imperial NHS Healthcare, London,; 5 Wexham Park Hospital and; 6 Heatherwood Hospital, Frimley Health NHS Foundation Trust, Berkshire, UK

**Keywords:** Tuberculosis, latent M. tuberculosis infection, cellular immune signatures, risk stratification, diagnostic.

## Abstract

**Background.:**

Recently acquired and remotely acquired latent *Mycobacterium tuberculosis* infection (LTBI) are clinically indistinguishable, yet recent acquisition of infection is the greatest risk factor for progression to tuberculosis in immunocompetent individuals. We aimed to evaluate the ability of cellular immune signatures that differ between active tuberculosis and LTBI to distinguish recently from remotely acquired LTBI.

**Methods.:**

Fifty-nine individuals were recruited: 20 had active tuberculosis, 19 had recently acquired LTBI, and 20 had remotely acquired LTBI. The proportion of mycobacteria-specific CD4^+^ T cells secreting tumor necrosis factor α (TNF-α) but not interferon γ or interleukin 2 which had a differentiated effector phenotype (TNF-α–only T_EFF_), and the level of CD27 expression on IFN-γ–producing CD4^+^ T cells, were detected by flow cytometry.

**Results.:**

The TNF-α–only T_EFF_ signature was significantly higher in the group with recently acquired LTBI, compared with the group with remotely acquired LTBI (*P* < .0001), and it discriminated between these groups with high sensitivity and specificity, with an area under the curve of 0.87. Two signatures incorporating CD27 expression did not distinguish between recently and remotely acquired LTBI. Interestingly, the TNF-α–only T_EFF_ signature in participants with recently acquired LTBI was more similar to that in participants with tuberculosis than that in participants with remotely acquired LTBI, suggesting that recently acquired LTBI is immunologically more similar to tuberculosis than remotely acquired LTBI.

**Conclusions.:**

These findings reveal marked biological heterogeneity underlying the clinically homogeneous phenotype of LTBI, providing a rationale for immunological risk stratification to improve targeting of LTBI treatment.

## Summary

Persons with latent *Mycobacterium tuberculosis* infection (LTBI) vary by their risk of progression to tuberculosis but are clinically indistinguishable. We identified a cellular immune signature that correlates strongly with time since exposure, providing a rationale for immunological risk stratification to improve targeting of LTBI treatment.

Identification of latent *Mycobacterium tuberculosis* infection (LTBI) and prevention of subsequent progression to tuberculosis is the cornerstone of tuberculosis control in most high-income, low-incidence countries (such as those in Europe and the United States) [1–3]. However, preventive treatment is long (≥3 months) and can have significant side effects. It is therefore a clinical imperative to identify persons at greatest risk of progression from LTBI to tuberculosis. Among immunocompetent individuals (who account for the vast majority of LTBI and tuberculosis cases globally), the single strongest clinical risk factor for progression from LTBI to tuberculosis is time since infection [4], with the risk being much higher in the first 2 years after infection (1.5% annual risk) and declining dramatically thereafter (approximately 0.1% annual risk) [5, 6]. However, distinguishing recently acquired from remotely acquired LTBI is very challenging, time-consuming, and frequently unreliable in routine clinical practice.

A blood-based biomarker that could risk-stratify persons with LTBI by distinguishing recently acquired from remotely acquired infection would greatly enhance LTBI screening and contact investigations and reduce the number of people unnecessarily treated. Several studies have identified cellular immune subsets that differ in proportions between patients with tuberculosis and those with LTBI [7–10]. We previously identified differences in *Mycobacterium tuberculosis*–specific T-cell populations and cytokine secretion profiles in patients with tuberculosis versus those with LTBI. Specifically, we demonstrated that the proportion of purified protein derivative–specific CD4^+^ T cells secreting tumor necrosis factor α (TNF-α) but not interferon γ (IFN-γ) or interleukin 2 (IL-2) which had a differentiated effector memory (CD45RA^−^CCR7^−^CD127^−^) phenotype (TNF-α–only T_EFF_) was able to distinguish tuberculosis from LTBI [8]. Others have also demonstrated that measuring the levels of CD27 expression on *M. tuberculosis–*specific cytokine-producing CD4^+^ T cells has the potential to distinguish between tuberculosis and LTBI [9–11]. For example, the TAM-TB assay, which evaluates the ratio of the median fluorescence intensity of CD27 within the whole CD4^+^ T-cell population to that of CD27 in the *M. tuberculosis–*specific IFN-γ^+^ CD4^+^ T cells has been shown to distinguish between tuberculosis in LTBI in children and adults [10]. Although it is increasingly believed that LTBI may be biologically heterogeneous, such assays and cellular immune profiling in general have not hitherto been used to dissect different epidemiological subgroups of LTBI that differ with respect to their risk of progression to tuberculosis.

We hypothesized that the long-term immune control that maintains host-pathogen equilibrium in remotely acquired established LTBI is not yet manifest in recently acquired LTBI where bacillary replication may initially be relatively uncontrolled, thereby resulting in a more differentiated effector T-cell phenotype. We therefore interrogated cellular immune signatures in clinically and epidemiologically precisely phenotyped subjects with LTBI who had clear and substantial differences in reported times since acquisition of *M. tuberculosis* infection.

## METHODS

### Study Participants and Recruitment

Participants were prospectively enrolled between February 2009 and May 2016 during routine National Health Service screening for tuberculosis or LTBI at one of the following National Health Service trusts in the United Kingdom: Imperial College Healthcare, Frimley Health, Bart’s Healthcare, and London Northwest. Participants were recruited under National Research Ethics Service approval (07/H0712/85 and 11/H0722/8), provided informed consent, and were aged ≥18 years. Individuals with known human immunodeficiency virus (HIV) infection were excluded; we did not routinely test all individuals in this cohort for HIV infection, but when testing had been previously clinically indicated, we were able to confirm that the test was negative.

### Grouping Criteria

Tuberculosis was confirmed by *M. tuberculosis*–positive culture of a sputum specimen (for pulmonary tuberculosis) or biopsy sample (for extrapulmonary tuberculosis). LTBI was confirmed by detection of *M. tuberculosis* infection (based on a positive result of an IFN-γ–release assay [ie, TSPOT.TB and/or QuantiFERON-Gold] and/or a tuberculin skin test), as well as the absence of symptoms of tuberculosis or clinical signs of tuberculosis on chest radiography. A positive tuberculin skin test result was defined as an induration diameter of ≥5 mm for BCG-unvaccinated individuals and ≥15 mm for BCG-vaccinated individuals [3]. Detailed demographic and epidemiological data were collected for each participant by a tuberculosis nurse dedicated to the project, using a standardized case report form to estimate the likely time since *M. tuberculosis* exposure and infection.

Individuals with LTBI were grouped according to whether they had recently or remotely acquired LTBI (Supplementary Table 1). Individuals with LTBI who did not meet the stringent criteria for either group (eg, those for whom there was potential exposure between 6 months and 2 years before recruitment) were excluded from the study.

#### Recently Acquired LTBI

Individuals were considered to have recently acquired LTBI if they had come in to close contact with a confirmed case of tuberculosis within 6 months prior to recruitment and were identified through contact investigations according to United Kingdom national guidelines [3]. For this group, the time since the most recent exposure was established through a detailed questionnaire and was used as the estimated time since infection.

#### Remotely Acquired LTBI

Individuals were considered to have remotely acquired LTBI if they were born in a tuberculosis-endemic country or an era of high tuberculosis incidence (>40 cases/100000), had lived in a country of low incidence for >2 years before recruitment, and had had no known contact with a tuberculosis case since emigrating or since the time at which the country of birth was no longer considered to have a high tuberculosis prevalence. For those who had emigrated from regions of high tuberculosis incidence but had had no known tuberculosis contact since emigration, the time since entry to a country with a low incidence of tuberculosis was used as a proxy for the time since exposure and infection; this represents the minimum possible time since infection with *M. tuberculosis*.

### Laboratory Measurement of Immunological Signatures

Peripheral blood mononuclear cell samples from individuals were processed, stored, stimulated, and analyzed for 3 published *M. tuberculosis–*specific cellular immune signatures as described previously [8–10], with minor modifications (Supplementary Materials). Laboratory researchers conducting the experiments were blind to the patient groups, and each experiment was designed to include individuals from differing patient groups to avoid batch effects.

### Statistical Analysis

Statistical analyses of immunological data from clinical groups were conducted using GraphPad Prism (version 6) and R statistical programming language (version 3.1.3) [12]. Further details are available in the Supplementary Materials.

## RESULTS

### Cohort Characteristics

Fifty-nine participants were recruited and assigned to the untreated tuberculosis group (n = 20), the untreated recently acquired LTBI group (n = 19), or the untreated remotely acquired LTBI group (n = 20), based on strict predefined criteria (see Methods and Supplementary Table 1). Patient demographic and clinical characteristics are presented in Table 1; there were no significant differences in age or sex proportions among groups.

**Table 1.** Demographic Characteristics of the Study Cohort, Overall and by Clinical Group

Characteristic	Total (n = 59)	Tuberculosis (n = 20)	Recently Acquired LTBI (n = 19)	Remotely Acquired LTBI (n = 20)
Age, y, median (range)	38 (21–78)	41.8 (22–78)	37 (21–70)	34 (21–73)
Male sex	34 (57.6)	14 (70)	13 (68)	7 (35)
Region of birth				
Western Europe	22 (37.3)	4 (20)	8 (42.1)	4 (20)
Eastern Europe	4 (6.8)	0 (0)	4 (21.1)	0 (0)
Middle East and North Africa	2 (3.4)	1 (5)	0 (0)	1 (5)
Sub-Saharan Africa	10 (16.9)	2 (1)0	3 (15.8)	5 (25)
Indian subcontinent	11 (18.6)	9 (45)	2 (10.5)	6 (30)
Central and Southeast Asia	7 (11.9)	3 (15)	1 (5.3)	3 (15)
Latin America and Caribbean	3 (5.1)	1 (5)	1 (5.3)	1 (5)
Ethnicity				
White British	5 (8.5)	1 (5)	3 (15.8)	1 (5)
White, other	9 (15.3)	2 (10)	5 (26.3)	2 (10)
Middle Eastern/Arabic	2 (3.4)	1 (5)	0 (0)	1 (5)
Pakistani	4 (6.8)	0 (0)	2 (10.5)	2 (10)
Indian	16 (27.1)	10 (50)	2 (10.5)	4 (20)
Chinese	1 (1.6)	0 (0)	0 (0)	1 (5)
Bangladeshi	1 (1.6)	0 (0)	1 (5.3)	0 (0)
Asian, other	6 (10.2)	3 (15)	1 (5.3)	2 (10)
Black African	9 (15.3)	1 (5)	2 (10.5)	6 (30)
Black Caribbean	4 (6.8)	2 (10)	2 (10.5)	0 (0)
Hispanic/South American	2 (3.4)	0 (0)	1 (5.3)	1 (5)
Occupation				
Health or social care	9 (15.3)	2 (10)	1 (5.3)	6 (30)
Non–health or non–social care	27 (45.8)	11(55)	11 (57.9)	5 (25)
Retired	5 (8.5)	2 (10)	2 (10.5)	1 (5)
Student	5 (8.5)	0 (0)	1 (5.3)	4 (20)
Unemployed	11 (18.6)	4 (20)	4 (21.1)	3 (15)
Unknown	2 (3.4)	1 (5)	0 (0)	1 (5)
TST result				
Positive	27 (45.8)	8 (40)	13 (68.4)	6 (30)
Negative	3 (5.1)	0 (0)	1 (5.3)	2 (10)
Not tested	29 (49.2)	12 (60)	5 (26.3)	12 (60)
IGRA result				
Positive	47 (79.7)	14 (70)	14 (73.7)	19 (95)
Negative	4 (6.8)	1 (5)	2 (10.5)^a^	1 (5)^a^
Not tested	8 (13.5)	5 (25)	3 (15.8)^a^	0 (0)
BCG receipt				
Yes	48 (81.4)	18 (90)	16 (84.2)	14 (70)
No	9 (15.3)	0 (0)	3 (15.8)	6 (30)
Unknown	2 (3.4)	2 (10)	0 (0)	0 (0)

Data are no. (%) of patients, unless otherwise indicated.

Abbreviations: IGRA, interferon γ–release assay; LTBI, latent *Mycobacterium tuberculosis* infection; TST, tuberculin skin test.

^a^The patient(s) had a positive TST result.

Individuals in the recently acquired LTBI group were contacts of patients with tuberculosis and were recruited as part of contact tracing, as per United Kingdom national guidelines [3]. Immune responses to *M. tuberculosis* in individuals with recently and remotely acquired LTBI were compared using the following 3 *M. tuberculosis–*specific cellular immune signatures, which have previously been shown to differ between tuberculosis and LTBI: the TNF-α–only T_EFF_ signature [8], the ratio of the median fluorescence intensity of CD27 expression on CD4^+^ T cells to that of CD27 expression on IFN-γ^+^ CD4^+^ T cells (determined by the TAM-TB assay [10]), and the proportion of IFN-γ^+^ CD4^+^ T cells with a CD45RA^−^CD27^−^ or CD45RA^−^CD27^+^ phenotype [9]. The gating strategies used for each signature are shown in Supplementary Figure 1.

### The TNF-α–Only T_EFF_ Signature Can Distinguish Recently Acquired From Remotely Acquired LTBI

**Figure 1.**
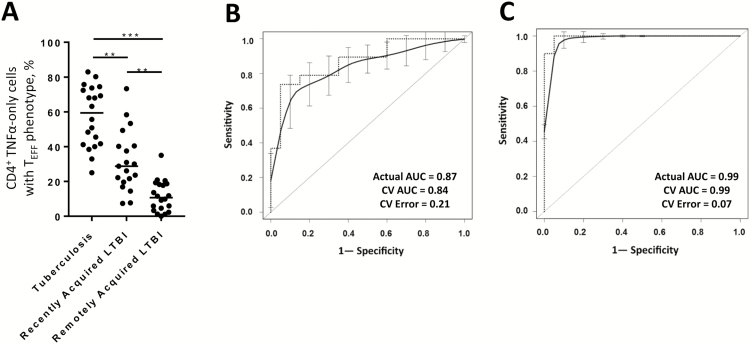
The TNF-α–only T_EFF_ signature, characterized by the proportion of CD4^+^ T cells secreting tumor necrosis factor α (TNF-α)-only which have a differentiated effector phenotype, in patients with tuberculosis, recently acquired latent *Mycobacterium tuberculosis* infection (LTBI), or remotely acquired LTBI. The proportion of purified protein derivative–specific TNF-α–only (not producing interferon γ or interleukin 2) producing CD4^+^ T cells with a differentiated effector memory (CD45RA^−^CCR7^−^CD127^−^) phenotype (TNF-α–only T_EFF_) was enumerated by flow cytometry for individuals with tuberculosis, recently acquired LTBI, or remotely acquired LTBI. *A*, Individual proportions of these cells in patients with tuberculosis (n = 20), recently acquired LTBI (n = 19), or remotely acquired LTBI (n = 20) is shown. **P < .01 and ***P < .001, by the Kruskal–Wallis test with the Dunn post hoc test for multiple comparisons. *B* and *C*, Receiver operating characteristic (ROC) curves demonstrating the ability of the TNF-α–only T_EFF_ signature to distinguish recently acquired from remotely acquired LTBI (*B*) and tuberculosis from remotely acquired LTBI (*C*). For the ROC curves, heavy dashed lines show the true performance of the TNF-α–only T_EFF_ signature; smooth lines with confidence intervals show the performance of the signature after 10-fold cross-validation was applied. The area under the curve (AUC) for the actual data sets (actual AUC) and after cross-validation (CV) analysis are shown, as well as the CV error rate.

Evaluation of the TNF-α–only T_EFF_ signature revealed significant differences in the proportion of these cells between all groups, with individuals with tuberculosis displaying the largest proportions (median, 59.4%; interquartile range [IQR], 41%–73%), followed by those with recently acquired LTBI (median, 28.8%; IQR, 20%–40%) and those with remotely acquired LTBI (median, 10.7%; IQR, 4%–19%; Figure 1A). The range of the proportions of TNF-α–only T_EFF_ cells in the recently acquired LTBI group in the current study was far wider than in the remotely acquired LTBI group and is similar to that in the tuberculosis patient group (Figure 1A). Receiver operating characteristic (ROC) curve analysis using the raw data gave an area under the curve (AUC) of 0.87, demonstrating that this signature was able to distinguish recently acquired from remotely acquired LTBI with a sensitivity of 89% (95% confidence interval [CI], 54.43%–93.95%) and a specificity of 65% (95% CI, 56.34%–94.27%) when a cutoff of >14.0% was used (Figure 1B). When a nested 10-fold cross-validation (CV) method was applied to the data set, the area under the curve (AUC) for distinguishing recently acquired from remotely acquired LTBI was 0.84, with a CV error rate of 0.21. The TNF-α–only T_EFF_ signature could almost completely distinguish between individuals with tuberculosis and those with remotely acquired LTBI, with an AUC of 0.99 revealed using either the raw data or after CV analysis (CV error rate, 0.7); comparison of these groups showed that a cutoff of >22.9% could distinguish between groups with a sensitivity of 100% (95% CI, 83.16%–100%) and a specificity of 95% (95% CI, 75.13%–99.87%; Figure 1C).

**Figure 2.**
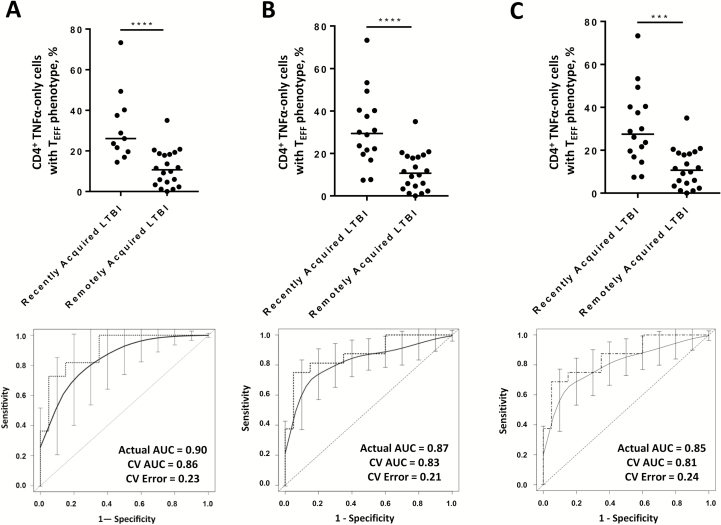
Sensitivity analyses for recently acquired versus remotely acquired latent *Mycobacterium tuberculosis* infection (LTBI), using the TNF-α–only T_EFF_ signature, characterized by the proportion of CD4^+^ T cells secreting tumor necrosis factor α (TNF-α) but not interferon γ or interleukin 2, which have a differentiated effector (CD45RA^−^CCR7^−^CD127^−^) phenotype. A direct comparison of the ability of the proportion of purified protein derivative–specific CD4^+^ T cells with a TNF-α–only T_EFF_ signature to distinguish recently acquired from remotely acquired LTBI (n = 20) was performed using only the recently acquired LTBI where there was no possible remote exposure (ie, those who had not lived in a country with high endemicity for TB) (n = 11; *A*), only recently acquired LTBI cases for which the index case was smear or culture positive for tuberculosis (n = 16; *B*), and only recently acquired LTBI cases for which the index case had pulmonary tuberculosis (n = 16; *C*). For each comparison, the upper panels show dot plots representing proportions of these cells for individuals in each group. ***P < .001 and ****P < .0001, by the Mann–Whitney *U* test. In the lower panels, receiver operating characteristic (ROC) curves of the percentage of TNF-α–only T_EFF_ cells demonstrate the ability of the signature to distinguish recently acquired from remotely acquired LTBI. The heavy dashed lines represent the true performance of the TNF-α–only T_EFF_ signature by using the cohort data sets, while the smooth lines with confidence intervals represent the performance of the signature after 10-fold cross-validation (CV) was applied. The area under the curve (AUC) for the actual data set is shown (actual AUC) in each ROC curve, as well as after CV analysis; the CV error rate is also given. Although the ethnic composition of the strict recently acquired LTBI group presented in panel *A* was changed to include a larger proportion of individuals who were white, it remained the case that within this subgroup, there was no significant differences in the TNF-α–only T_EFF_ signature between ethnic groups.

All individuals with recently acquired LTBI were recruited as part of contact tracing as per national guidelines; however, in some cases it may be that either their infection was actually acquired remotely or that the identified index case was not actually infectious (therefore, the reported time since infection may not be accurate). To address these issues, we performed sensitivity analyses where individuals for whom the identified recent exposure may not have been the cause of their infection were excluded (Supplementary Methods). In each of these more stringent analyses, the performance of the signature for discriminating between recently acquired LTBI and remotely acquired LTBI was unaffected (Figure 2).

Subsequent follow up since recruitment, using the London Tuberculosis Register, indicated that none of the study participants with LTBI had developed tuberculosis after a follow-up period ranging from 1 month to 6.5 years (median, 22 months). This is unsurprising given that the majority (31 of 39 [79%]) commenced LTBI treatment after recruitment into the study.

### Cellular Immune Signatures Incorporating CD27 Do Not Distinguish Between Recently Acquired and Remotely Acquired LTBI

**Figure 3.**
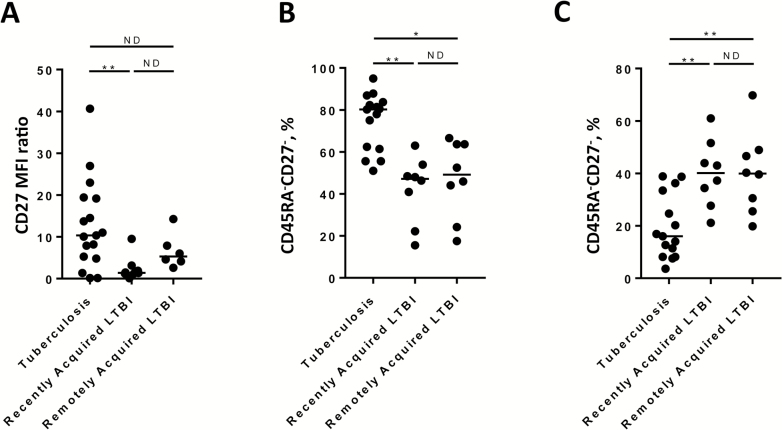
Comparison of signatures incorporating CD27 expression between patients with recently acquired latent *Mycobacterium tuberculosis* infection (LTBI) and those with remotely acquired LTBI. Cellular signatures that incorporate CD27 expression were compared between individuals with tuberculosis, those with recently acquired LTBI, and those with remotely acquired LTBI. *A*, The ratio of median fluorescence intensity (MFI) of CD27 in the whole CD4^+^ T-cell population to the MFI of CD27 of the purified protein derivative (PPD)–specific interferon γ–expressing (IFN-γ^+^) CD4^+^ T-cell population was calculated (by the TAM-TB assay [10]) and compared between groups among patients who satisfied the cutoff criteria for this assay (17 in the tuberculosis group, 8 with recently acquired LTBI, and 6 with remotely acquired LTBI). The proportions of purified protein derivative–specific CD4^+^ IFN-γ^+^ cells with a CD45RA^−^CD27^−^ (*B*) or CD45RA^−^CD27^+^ (*C*) phenotype were calculated and compared between groups (15 in the tuberculosis group, 8 with recently acquired LTBI, and 8 with remotely acquired LTBI). *P < .05 and **P < .01, by the Kruskal–Wallis test with the Dunn post hoc test for multiple comparisons. ND, no difference.

*M. tuberculosis–*specific immune signatures that incorporate CD27 have shown promise in distinguishing between tuberculosis and LTBI. CD27 staining was included in the flow cytometry assays for 17 cases of tuberculosis, 8 cases of recently acquired LTBI, and 8 cases of remotely acquired LTBI. *M. tuberculosis–*specific IFN-γ^+^ responses were analyzed for 2 such signatures—the change in intensity of CD27 expression (determined by the TAM-TB assay) and the proportion of both CD45RA^−^CD27^−^ and CD45RA^−^CD27^+^ phenotypes—and these signatures were compared between groups. These analyses identified significant differences between tuberculosis as compared to either recently acquired LTBI alone (in the case of TAM-TB assay) or both recently acquired and remotely acquired LTBI (when proportions of CD45RA^−^CD27^−^ or CD45RA^−^CD27^+^ phenotypes were compared; Figure 3). However, these signatures were not significantly different between recently acquired and remotely acquired LTBI and therefore would not be useful for discriminating between these groups.

## DISCUSSION

We have demonstrated that the TNF-α–only T_EFF_ cellular immune signature associates closely with the single strongest risk factor for progression of LTBI to tuberculosis in immunocompetent adults: time since infection. Previous studies have identified several cellular immune signatures that differ between the clinically distinct states of tuberculosis and asymptomatic LTBI [8, 11, 13]. However, none have described distinct immunological signatures within LTBI that correlate with a well-defined risk factor for progression. We have found evidence that LTBI, while clinically homogeneous, with an absence of symptoms and normal chest radiographic findings, is immunologically heterogeneous, with the reported time since acquisition being a major contributing factor to that heterogeneity.

We previously demonstrated that the TNF-α–only T_EFF_ signature distinguished tuberculosis from LTBI with high sensitivity and specificity, with the proportion of these cells being significantly higher in patients with tuberculosis than in patients with LTBI, including patients with HIV coinfection [8]. Here we show that the proportion of these cells is significantly higher during recently acquired LTBI as compared to remotely acquired LTBI. Consistent with this novel observation, the difference in the TNF-α–only T_EFF_ signature between tuberculosis and LTBI reported by Pollock et al was derived from a cohort of subjects with LTBI who had mostly acquired their infection remotely [8]. We were therefore able to validate the use of this signature when discriminating tuberculosis from remotely acquired LTBI, demonstrating consistently high performance in 2 independent studies and after a CV approach was used in the current data set. Thus, a combined cell surface loss of expression of CD45RA, CCR7, and CD127 on *M. tuberculosis–*specific CD4^+^ T cells is associated with both tuberculosis and recently acquired LTBI but not with remotely acquired LTBI.

Loss of CD27 expression on *M. tuberculosis*–specific IFN-γ^+^ CD4^+^ T cells has been shown to be associated with tuberculosis when compared to LTBI in multiple studies. In this study, 2 signatures incorporating such measurements did not differ significantly between recently acquired and remotely acquired LTBI in our cohort. This indicates that loss of CD27 expression on *M. tuberculosis–*specific CD4^+^ T cells, while associated with clinical disease [9–11], is not related to time since infection in patients with LTBI. However, we did demonstrate that both the TAM-TB assay and the proportions of CD45RA^−^CD27^−^ or CD45RA^−^CD27^+^ IFN-γ^+^ CD4^+^ T cells differed between patients with tuberculosis and those with LTBI. We thus independently validated the ability of these signatures for discriminating tuberculosis from LTBI [9, 10], supporting their reproducibility.

Very few other studies to date have subclassified individuals with LTBI by using time since infection, when exploring immunological signatures. One study demonstrated that an innate immune cell subset, myeloid-derived suppressor cells (which are known to suppress CD4^+^ T-cell function), was significantly increased during both tuberculosis and recently acquired LTBI, compared with treated tuberculosis and remotely acquired LTBI [14]. This is consistent with our findings that recently acquired LTBI appears immunologically comparable to tuberculosis.

The range in the proportions of TNF-α–only T_EFF_ cells in the recently acquired LTBI group in the current study was far wider than range of those in the remotely acquired LTBI group, likely reflecting the relative heterogeneity in terms of the risk of progression that exists for patients with recently acquired LTBI, compared with the homogeneity of responses and very low risk of progression for patients with remotely acquired LTBI [4, 6]. Studies involving *M. tuberculosis–*infected macaques have demonstrated that the total pulmonary bacterial burden, as well as the *M. tuberculosis* burden within specific lesions in the lung, are higher in both recent infection and clinically manifest active disease, compared with established (ie, remotely acquired) LTBI [15]. These microbiological data from the lungs of infected macaques support our immunological findings that recently acquired LTBI is more similar than remotely acquired LTBI to tuberculosis. We speculate that patients in the LTBI group who have a higher TNF-α–only T_EFF_ response, which overlaps most with the tuberculosis group, may be more likely to progress to tuberculosis. However, given that most of our cohort subsequently received LTBI treatment, we were unable to test this hypothesis in this study. Once validated in an independent cohort, a test measuring the TNF-α–only T_EFF_ signature could be further developed as a second-line test to an IFN-γ–release assay, to substantially reduce the proportion of patients with remotely acquired LTBI who are offered treatment, while still identifying all patients with recently acquired infection.

The potential for particular mycobacterial antigens to elicit differential immune responses in distinct subgroups of individuals with LTBI has previously been investigated. Although differential responses to putative “latency-associated” antigens have been identified [16–18], these have not hitherto been shown to correlate with progression to tuberculosis, the clinical risk of progression, or any other clinical parameter. A recent study in individuals with long-standing LTBI identified some immunological differences that appeared to correlate with an unvalidated online risk calculator [19]. However, since the study population had been infected for an average of 12 years and there were no individuals with recently acquired LTBI [19], the participants were neither at risk of progression nor eligible for LTBI treatment according to guidelines. The study identified differences in the measured immunological parameters between treated and untreated LTBI, which has been shown previously for other immune signatures during both tuberculosis [20–22] and LTBI [23].

Our study has some limitations. Although our cohort is defined by high-quality and detailed demographic, clinical, and epidemiological data, coupled with stringent criteria for recently acquired versus remotely acquired LTBI, one can never be certain precisely when a given individual acquired infection, and we cannot control for recall bias. We therefore chose to compare 2 subgroups of patients with LTBI with considerably different reported times since exposure to *M. tuberculosis*, based on the extensive epidemiological information we systematically collected for this study (not as part of routine practice), and we excluded those who did not meet our stringent predefined criteria. Despite this, we cannot be certain that some individuals in the recently acquired LTBI group had not actually acquired their current infection remotely. To address this problem, we performed an even stricter analysis, in which patients with recently acquired LTBI who had any risk of having been exposed at a different time to the identified recent exposure were excluded, with no impact on the performance of the signature.

All of the signatures presented here are measures of immune responses to purified protein derivative, a crude mixture of *M. tuberculosis* antigens that elicits strong, predominantly CD4^+^ T-cell cytokine responses. Although this confers good sensitivity for *M. tuberculosis* infection in the immunological assays used here, the specificity is impaired because of cross-reactivity in BCG-vaccinated individuals. Thus, in clinical practice, these signatures would likely be used as a risk-stratification tool for subjects with positive results of the ESAT-6/CFP-10–based IFN-γ–release assay, which does reliably distinguish individuals with *M. tuberculosis* infection from recipients of BCG vaccine [24]. Hence, the antigenic cross-reactivity of purified protein derivative used in our immune signatures would not be a problem in clinical practice. Staining for CD27 was not included for all experiments performed in this study. Therefore, the sample size may have lacked sufficient statistical power to detect differences in CD27 expression on *M. tuberculosis–*specific CD4^+^ T cells between patients with recently acquired LTBI and those with remotely acquired LTBI.

To validate our findings, a larger study in an independent population is now required before this test can be considered as a screening test for recently acquired LTBI. Ultimately, quantification of the forward risk associated with the respective immune signatures will require a long-term prospective longitudinal cohort study of individuals with LTBI followed up to assess for progression to tuberculosis and correlation with baseline immune signatures. In this study, we were able to evaluate 3 immune signatures that assess the memory and maturation phenotype of *M. tuberculosis–*specific T cells and that have previously been shown to differentiate tuberculosis and LTBI.

Our findings represent a significant advance on current tests of *M. tuberculosis* infection, which cannot differentiate recently acquired from remotely acquired LTBI [25]. Although the clinical unmet need for a test to risk-stratify persons with LTBI and thereby better target preventive treatment is large and urgent [26], the notion that immunological markers might distinguish recently acquired from remotely acquired LTBI is relatively novel. Our findings suggest that an immune-based test could be used to differentiate between different types of LTBI and, if validated in subsequent larger longitudinal studies, could usefully risk-stratify persons with LTBI for targeted preventive treatment.

## Supplementary Data

Supplementary materials are available at *The Journal of Infectious Diseases* online. Consisting of data provided by the authors to benefit the reader, the posted materials are not copyedited and are the sole responsibility of the authors, so questions or comments should be addressed to the corresponding author.

## Supplementary Material

Supplementary Materials

## Notes

***Acknowledgments.*** We thank the patients, for their participation in this study; Luis Berrocal-Almanza (NIHR HPRU and Imperial College London), for his critique in the manuscript and assistance in the interpretation of the data; Helen Piotrowski and Hiromi Uzu (Imperial College London) and Robert N Davidson (Northwick Park Hospital), for their role in recruitment of patients into the study; and Mica Tolosa-Wright (Imperial College London), for her technical support.

A. H., H. W., S. H. K., O. M. K., and A. L. participated in the conception/design of the study. S. M., S. B., A. B., H. K., O. M. K., and A. L. were involved in participant recruitment and sample collection. A. H., H. W., and S. H. K. performed the experimental work, while A. H., H. W., U. N., P. B., and A. L. were involved in the analysis and interpretation of the data. A. H. and H. W. prepared the data for publication and drafted the initial manuscript, but all authors contributed to the drafting and revision of the manuscript.

***Disclaimer.*** The funders played no role other than financial support in this work or in the preparation of the manuscript. The views expressed are those of the author(s) and not necessarily those of the NHS, the NIHR, the Department of Health or Public Health England.

***Financial support.*** This work was supported by the United Kingdom National Institute for Health Research Health Protection Research Unit (NIHR HPRU) in Respiratory Infections, as well as a Wellcome Trust Senior Clinical Research Fellowship and a NIHR Senior Investigator Award (both awarded to A. L.).

***Potential conflicts of interest.*** A. L. has issued several patents in relation to immunodiagnostics for tuberculosis. The ESAT-6/CFP-10 ELISpot was commercialized by an Oxford University spin-out company (Oxford Immunotec, Abingdon, United Kingdom) from which Oxford University and A. L. entitlement to royalties. All authors have submitted the ICMJE Form for Disclosure of Potential Conflicts of Interest. Conflicts that the editors consider relevant to the content of the manuscript have been disclosed.
